# Compressive electron backscatter diffraction imaging

**DOI:** 10.1111/jmi.13379

**Published:** 2025-01-11

**Authors:** Zoë Broad, Alex W. Robinson, Jack Wells, Daniel Nicholls, Amirafshar Moshtaghpour, Angus I. Kirkland, Nigel D. Browning

**Affiliations:** ^1^ Department of Mechanical, Materials and Aerospace Engineering University of Liverpool Liverpool UK; ^2^ SenseAI Innovations Ltd. Liverpool UK; ^3^ Correlated Imaging Group, Rosalind Franklin Institute Harwell Science and Innovation Campus Didcot UK; ^4^ Department of Materials University of Oxford Oxford UK

**Keywords:** compressive sensing, electron backscatter diffraction, EBSD, SEM

## Abstract

Electron backscatter diffraction (EBSD) has developed over the last few decades into a valuable crystallographic characterisation method for a wide range of sample types. Despite these advances, issues such as the complexity of sample preparation, relatively slow acquisition, and damage in beam‐sensitive samples, still limit the quantity and quality of interpretable data that can be obtained. To mitigate these issues, here we propose a method based on the subsampling of probe positions and subsequent reconstruction of an incomplete data set. The missing probe locations (or pixels in the image) are recovered via an inpainting process using a dictionary‐learning based method called beta‐process factor analysis (BPFA). To investigate the robustness of both our inpainting method and Hough‐based indexing, we simulate subsampled and noisy EBSD data sets from a real fully sampled Ni‐superalloy data set for different subsampling ratios of probe positions using both Gaussian and Poisson noise models. We find that zero solution pixel detection (inpainting un‐indexed pixels) enables higher‐quality reconstructions to be obtained. Numerical tests confirm high‐quality reconstruction of band contrast and inverse pole figure maps from only 10% of the probe positions, with the potential to reduce this to 5% if only inverse pole figure maps are needed. These results show the potential application of this method in EBSD, allowing for faster analysis and extending the use of this technique to beam sensitive materials.

## 1 INTRODUCTION

**FIGURE 1:**
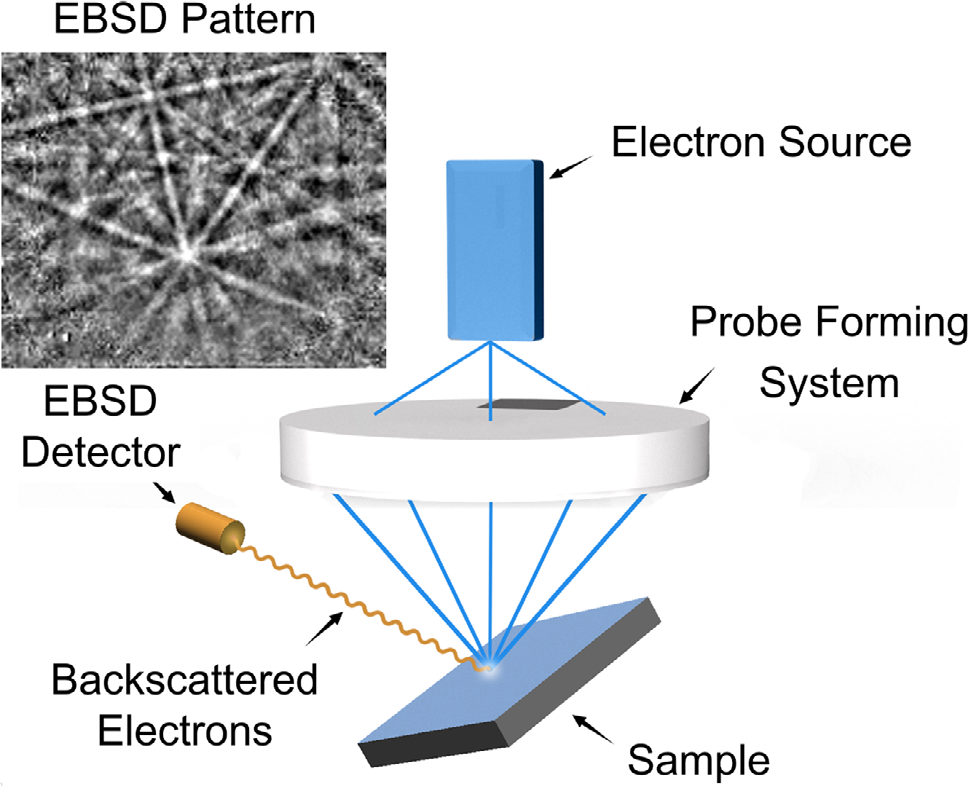
**Operating principles of EBSD imaging**. A convergent electron beam is raster scanned across the sample. Backscattered electrons form a pair of cones which intersect the phosphor screen, allowing the EBSD pattern to be read by the detector.

Electron backscatter diffraction (EBSD) is a scanning electron microscopy (SEM) technique, providing important crystallographic information about the sample such as crystal orientation and grain size.^1^ Figure 1 shows the typical instrument geometry, where an EBSD pattern (EBSP) is formed from an electron beam incident on a crystal plane of a highly tilted sample.^1^

Conventionally, an EBSD data set is acquired by scanning an electron beam across the surface of a sample in a raster scan, acquiring an EBSP at every position in the scan, that is, at each probe location. The measured EBSPs form a 4‐dimensional (4D) data set.^1^

**FIGURE 2:**
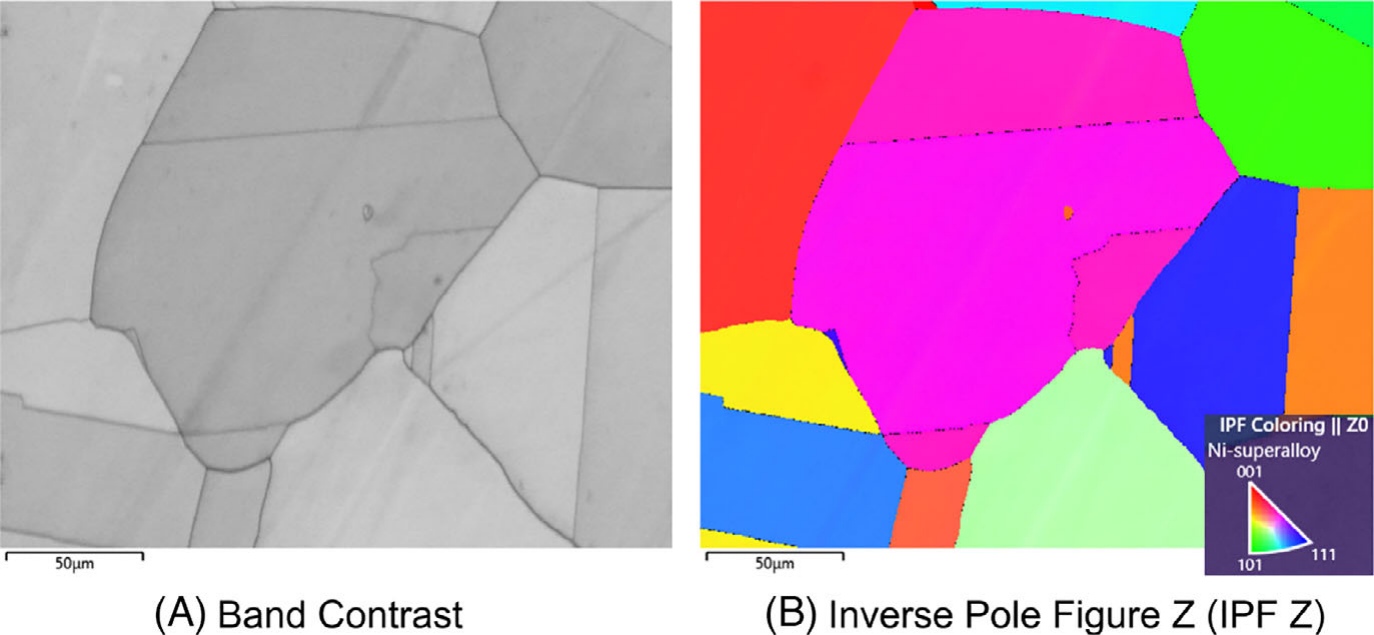
**Example EBSD maps of Ni‐superalloy**. EBSD maps provide crystallographic information about the sample such as (A) the locations of grain boundaries or (B) crystal orientation.

EBSPs contain information relating to crystal structure, and this information is extracted by indexing – that is the process of identifying the crystal phase and orientation from each EBSP. Multiple methods exist to allow this process to be performed automatically.^2^, ^3^, ^4^, ^5^, ^6^ Each EBSP corresponds to a single probe position in the sample and therefore to a single pixel in the indexed map. Each pixel in this map then represents some information gained from each EBSP. Band contrast and inverse pole figure (IPF) maps are two common types of EBSD maps, which are chosen as the focus of study in this work (Figure 2).

Band contrast maps are a measure of EBSP quality based on the contrast between the intensity of the Kikuchi bands compared to the intensity of the background in an EBSP. At grain boundaries and defects, the contrast is lower due to the superimposition of EBSPs from all grain boundaries in the interaction volume.^7^ This gives rise to a low indexing value in the band contrast map, producing dark pixels at the grain boundaries. This provides additional crystallographic information to be obtained compared to conventional secondary/backscattered electron imaging due to this mechanism.^8^ Other quality maps, for example, image quality and band slope maps provide similar information, and are expected to give equivalent results to those demonstrated here since they have the same image properties. Orientation maps such as an IPF map are a measure of the relative crystal orientation at each probe position, for example IPF Z map, also called a normal direction map, in Figure 2. In an IPF map the colour of each point corresponds to the crystallographic direction with regards to a Cartesian axis, that is, the *Z* axis in the case of the IPF Z map.

Despite significant advances in EBSD imaging, currently its applications remain limited due to, for example, sample preparation. Since a perfectly flat surface is needed for quality measurements, complex samples (such as geological samples or samples with many phases where there may be low signal) and detector speed can limit quality and/or quality of a data set. The fastest detectors currently on the market can reach speeds of over 6000 patterns per second^9^ but this depends on the sample and quality needed.

### 1.1 Contributions

**FIGURE 3:**
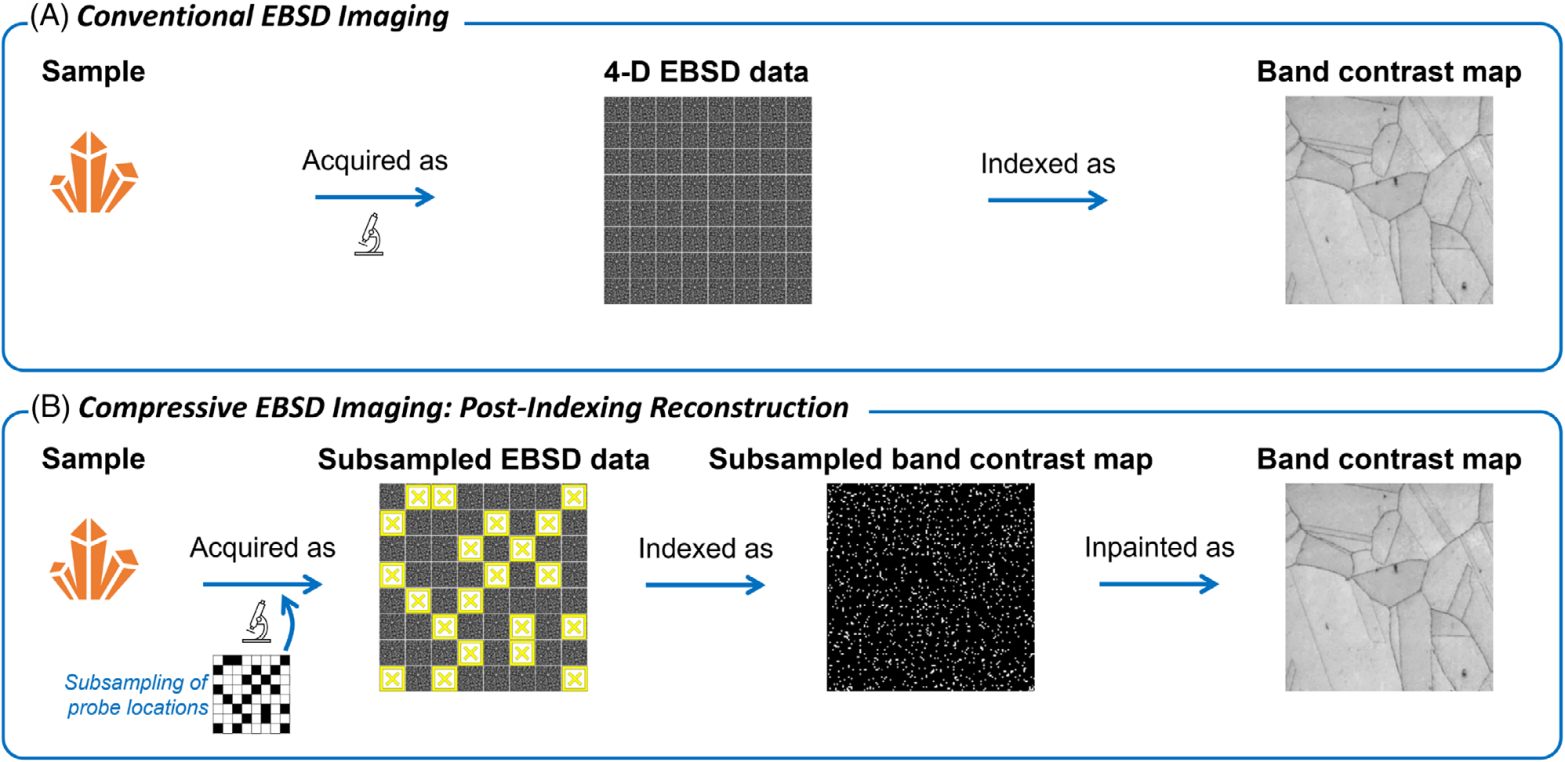
**Conventional versus compressive EBSD imaging**. Probe locations are subsampled and the EBSD patterns acquired are indexed. The incomplete maps formed can then be inpainted.

In this paper, we introduce an EBSD acquisition approach based on subsampling probe positions, which results in an incomplete 4D EBSD data set. Subsampling allows significantly faster data acquisition without any significant advances for any detector technology. Indexing such data, however, yields band contrast and IPF maps with missing values associated with unsampled probe positions. Our approach, as illustrated in Figure 3b, is to first index the subsampled EBSD data set and then to inpaint the resulting EBSD maps using beta‐process factor analysis (BPFA),^10^, ^11^ that is, a joint blind dictionary learning and sparse coding image inpainting method.

This paper aims to show a proof of concept, demonstrating the possibility of subsampling for EBSD. The focus of this paper is the parameters necessary for inpainting, since the optimisation of these parameters can be referenced directly to the 100% sampled ground truth. Without this first ground truth comparison, any change in the experimental acquisition is open to valid questions concerning its accuracy. As we have shown previously, the experimental parameters for subsampled acquisition in scanning transmission electron microscopy (STEM) are essentially unchanged from the fully sampled images other than acquiring fewer pixels.^12^, ^13^, ^14^ In future work, we will aim to demonstrate the limits of subsampling for EBSD, that is, how few pixels can be used to reconstruct images.

Here we consider only analyses where maps are produced to visualise sample texture, for example, where only a pattern quality or misorientation map is needed, rather than for the analysis of deformations where the maps produced require knowledge of neighbouring EBSPs. Additionally, it should be noted that the method applied here focuses on improving the speed of EBSD acquisition, since regardless of detector speed subsampling improves acquisition speed by acquiring fewer probe locations. It is anticipated that this could be most useful for identifying areas of interest, large area mapping, or for 3D EBSD where large quantities of data are being acquired. The latter also has the potential for targeted sampling to be applied, where the sampled datapoints are chosen based on the previous map.

Robustness to subsampling is first investigated here using a real data set acquired with high fluence. Robustness to noise is evaluated considering both a Gaussian and Poisson noise model; first without subsampling, testing the efficacy of the indexing procedure and then with subsampling, testing the efficacy of the inpainting method.

### 1.2 Related works

#### 1.2.1 Applications of compressive sensing in electron microscopy

Compressive sensing (CS) has seen successful applications in several modalities of electron microscopy for, for example, low dose or fast acquisition. The theory of CS consists of a set of conditions for recovering a signal from few direct or indirect measurements compared to the intrinsic dimension of that signal.^15^, ^16^ CS often relies on the fact that most data can be sparsely represented in an appropriate sparsity basis,^17^ or dictionary.

CS theory has been applied to multiple modes in electron microscopy. Scanning transmission electron microscopy (STEM) in particular has seen developments in this area, allowing for low dose acquisition to be realised.^12^, ^13^, ^18^, ^19^, ^20^, ^21^

CS has enabled fast and low‐dose 4D STEM systems.^22^, ^23^, ^24^ It has been demonstrated the application of CS allows sampling rates below the Nyquist criteria to be realised meaning that faster acquisition and lower beam dose can be implemented.^25^ STEM simulations have also benefited through the use of CS, allowing close to real time calculation of STEM images.^14^, ^26^ FIB‐SEM is another method that has seen CS applications, enabling a lower electron dose acquisition and reducing the long acquisition times.^27^, ^28^

#### 1.2.2 Alternative scanning strategies for EBSD imaging

The method described here is not the first alternative EBSD scanning strategy to be investigated for EBSD. A method of smart sampling, enabling the targeted sampling of grain boundaries was proposed by Yang et al.^29^ Another alternative strategy proposed by Tong et al.^30^ used the forward scattered electron (FSE) image acquired to identify grains, enabling single orientation measurements of each grain to be analysed. Closest to the method described in this paper is a method of determining grain size distribution proposed by Long et al. ^31^ which uses grid‐based, random and quasi‐random sampling for data acquisition. In this work the desired output is the grain size distribution, whilst we aim to reconstruct the EBSD maps.

The scanning strategies proposed by Yang et al and Long et al have the desired output of grain boundary information. As in our method, in Tong et al. a visualisation of texture is the key output. Our proposed method does not require any prior knowledge of the same, for example, through the FSE image, although this is a technique that could be investigated in the future.

#### 1.2.3 Camera subsampling

In EBSD imaging, a method of camera subsampling has been demonstrated by Wang et al.^32^ This method means that fewer pixels on the detector are acquired, demonstrating the significant speed benefits of subsampling. Our method differs by applying probe subsampling, which has the potential to be implemented in conjunction with camera subsampling, which would enable even faster acquisition.

#### 1.2.4 Cleaning strategies

Many methods are available for EBSD imaging, allowing samples with high levels of noise to be more accurately indexed, for example, by using information from neighbouring pixels, as in methods like grain dilation and neighbouring pattern averaging and reindexing (NPAR) or through alternative post‐processing of the full data set, such as nonlocal means pattern averaging and reindexing (NLPAR) or multivariate statistical analysis.^33^, ^34^, ^35^, ^36^ Although, as addressed in Brewer et al,^37^ there are risks of overprocessing the data sets which can result in misindexing or the formation of artefacts.

In this work we detect zero solution pixels, which are pixels where the EBSP has not been indexed by the indexing algorithm, and consider them to be an unsampled data point, which allows them to be inpainted in the same manner as the intentionally unsampled locations. Our method is most similar to that of grain dilation^33^ since it is based on the information of the neighbouring points. They key difference being that we clean the data as it is being inpainted.

## 2 CONVENTIONAL EBSD IMAGING AND ANALYSIS

In this section for convenience, we describe the conventional EBSD acquisition and analysis methods.

### 2.1 Acquisition model

A 4D EBSD data set is a collection of 2‐D EBSD patterns for every probe position. Consider a probe scanning $H_{p}\times W_{p}$ positions on a regular 2‐D grid with $N_{p}≔H_{p}W_{p}$ total probe positions. For each position of the probe, indexed by $l\in \{1,\text{…},N_{p}\}$, the detector records a 2‐D EBSD pattern over $H_{d}\times W_{d}$ pixels. We represent that EBSD pattern in its vectorised format $y_{l}\in R^{N_{d}}$, where $N_{d}≔H_{d}W_{d}$ is the total number of detector pixels.

Let $x\in R^{N_{p}}$ be a discretised and vectorised representation of an EBSD map, for example band contrast map or IPF map, where every pixel corresponds to one probe position. Without the need to specify an actual forward model,^4^, ^38^ we assume that every EBSD pattern $y_{l}$ is related to its corresponding pixel value in x through a sensing operator $A:R\mapsto R^{N_{d}}$. Mathematically, for every probe position $l\in \{1,\text{…},N_{p}\}$,

(1) $$y_{l}=A\left(x_{l}\right)\in R^{N_{d}},$$

where an additive or non‐additive noise model is included in A.

### 2.2 Analysis of complete data set

Once an EBSD data set has been acquired, additional processing, known as indexing, is necessary to extract useful crystallographic information from the EBSPs. Discussed here are different methods of indexing and their benefits.

Indexing refers to the identification of the crystal phase and orientation in the acquired EBSP. In Hough‐based indexing, EBSPs are indexed through a Hough transform, converting the bright bands of the EBSP into bright spots in Hough space.^3^, ^39^ This method is fast and allows for online indexing. However, the greatest disadvantages of the Hough transform are its sensitivity to noise and lower accuracy compared to alternative such as dictionary indexing.^4^ When noisy EBSPs are obtained, the hit rate (i.e. the percentage of EBSPs that have been indexed) of indexing based on Hough transform significantly decreases, resulting in incomplete EBSD maps.^4^

Dynamical simulations of EBSPs can be created based on a simplified model describing the interaction of the electrons in the sample, creating a spherical master EBSP of predicted reflections based on the sample properties.^1^ These simulations offer great benefits through alternative indexing techniques, such as dictionary and spherical indexing.

Dictionary indexing uses the master EBSP to create a set of templates followed by identifying the acquired EBSP orientation based on those templates.^4^ Although this method is less sensitive to noise, it is computationally expensive. The sizes of the dictionaries used increases as sample symmetry decreases, which further extends the computational cost and analysis times.^4^, ^40^

Spherical indexing uses a similar technique of EBSP matching. In this model, the EBSP is projected onto a sphere, allowing greater correlation in EBSP matching. This technique has shown promising results for noisy EBSPs, with increased speeds compared to dictionary indexing and greater robustness against noise than Hough transform‐based methods.^5^

The indexing approaches above treat one EBSP at a time. Therefore, regardless of the indexing method used, an estimation of the true EBSD map, that is, $\hat{x}\approx x$ is formed by indexing every EBSP denoted by an operator $\Delta :R^{N_{d}}\mapsto R$, that is for every EBSP indexed $l\in \{1,\text{…},N_{p}\}$,

(2) $${\hat{x}}_{l}=\Delta \left(y_{l}\right)=x_{l}+n_{l},$$

where $n_{l}$ represents indexing error. We note here that due to discrepancy sources in the forward operator A in Equation 1, the indexing process may fail. The pixels for which the indexing fails are referred to as *Zero‐Solution Pixels* (ZSPs). The indices of those pixels are collected in a ZSP set $\Omega_{\mathrm{zsp}}=\left\{l\in \left\{1,\text{…},N_{p}\right\}:\Delta \left(y_{l}\right)=0\right\}$.

## 3 PROPOSED METHOD: POST‐INDEXING RECONSTRUCTION OF SUBSAMPLED EBSD DATA

### 3.1 Acquisition model

The proposed compressive EBSD system operates by subsampling the probe positions as has been applied in the SEM by use of a scan generator, as shown in Ref. [28].

Let $\Omega \subset \{1\text{…},N_{p}\}$ be a subset of $\left|\Omega \right|=M_{p}$ probe positions. The ordering of the elements in Ω controls the trajectory of the scanning probe. Therefore, the acquisition model of compressive EBSD imaging is identical to Equation 2, but only for probe positions l∈Ω. Since the acquisition time of an EBSD data set is proportional to the number of scanned probe positions, by subsampling $M_{p}$ probe positions the acquisition time will be reduced by a factor of $M_{p}/N_{p}$ compared to the full acquisition. The total electron fluence is also reduced by subsampling probe positions. Our model supports any arbitrary subsampling strategy, such as Uniform Density Sampling (UDS)^27^ – that is, selecting a probe position uniformly at random, linehop^12^ – that is, subsampling at random the locations adjacent to the probe's default line trajectory, or dynamic sampling.^41^, ^42^

### 3.2 Analysis of an incomplete data set

Given a subsampled 4D EBSD data, an EBSD map $z\in R^{N_{p}}$ can be computed using an indexing operator Δ. Similar to conventional EBSD imaging, the indexing process follows Equation 2 for sampled probe locations. However, the value of an EBSD map for unsampled probe positions reads zero. Mathematically,

(3) $$z_{l}=\left\{\begin{array}{cc} {\hat{x}}_{l}, & \mathrm{if}\;l\in \Omega ,\\ 0, & \mathrm{if}\;l\notin \Omega . \end{array}\right.$$

From Equation 2, this becomes

(4) $$z=P_{\Omega }\left(x+n\right),$$

where $n≔{\left[n_{1},\text{…},n_{N_{p}}\right]}^{⊤}$ is a noise vector collecting indexing errors; and $P_{\Omega }\in {\left\{0,1\right\}}^{N_{p}\times N_{p}}$ is a mask operator of probe positions, such that ${\left(P_{\Omega }x\right)}_{l}=x_{l}$ if l∈Ω and ${\left(P_{\Omega }x\right)}_{l}=0$, otherwise.

We note that ZSPs result in zero values in vector z. Depending on the application, those ZSPs can be detected and treated as unsampled pixels, that is, $\Omega \leftarrow \Omega \cup \Omega_{\mathrm{zsp}}$. We revisit this scenario in Section 4.

### 3.3 Recovery of EBSD maps

Our goal in this section is to recover a high‐quality estimate of the EBSD map, that is, $\hat{z}\approx x$, from an incomplete EBSD map z using inpainting.

Inpainting is the recovery of an observation from fewer direct measurements. For image data, there are various inpainting methods based on, for example interpolation,^32^, ^43^ deep learning,^44^, ^45^ and sparse coding^46^, ^47^ approaches. In this work, we consider beta‐process factor analysis (BPFA): a joint dictionary learning and sparse coding algorithm from a sub‐sampled set of measurements.

BPFA operates on a patch‐wise basis. Given an observation z as in Equation 4, the image is broken down in to a set of $H_{\mathrm{op}}\times W_{\mathrm{op}}$ overlapping patches. The number of overlapping patches is thus $N_{\mathrm{patch}}=\left(H_{p}-H_{\mathrm{op}}+1\right)\left(W_{p}-W_{\mathrm{op}}+1\right)$. Each overlapping patch is then vectorised as $z_{p}\in R^{N_{\mathrm{op}}}$ with length $N_{\mathrm{op}}=H_{\mathrm{op}}W_{\mathrm{op}}$, forming a collection ${\left\{z_{p}\right\}}_{p=1}^{N_{\mathrm{patch}}}$ of all overlapping patches. Accordingly, the EBSD map x, noise vector n, and subsampling operator $P_{\Omega }$ are partitioned and form the collections ${\left\{x_{p}\right\}}_{p=1}^{N_{\mathrm{patch}}}$, ${\left\{n_{p}\right\}}_{p=1}^{N_{\mathrm{patch}}}$, and ${\left\{P_{\Omega_{p}}\right\}}_{p=1}^{N_{\mathrm{patch}}}$, respectively. With those definitions, the acquisition model of each overlapping patch is given by

(5) $$z_{p}=P_{\Omega_{p}}\left(x_{p}+n_{p}\right)\in R^{N_{\mathrm{op}}},\;\mathrm{for}\;p\in \left\{1,\text{…},N_{\mathrm{patch}}\right\}.$$

As required by BPFA, every patch of the EBSD map $x_{p}\in R^{N_{\mathrm{op}}}$ is assumed to have a sparse representation in a shared dictionary $D\in R^{N_{\mathrm{op}}\times K}$ of K atoms, that is for $p\in \{1,\text{…},N_{\mathrm{patch}}\}$,

(6) $$x_{p}=D\alpha_{p},\;\mathrm{with}\;{∥\alpha_{p}∥}_{0}\leq s,$$

where $\alpha_{p}\in R^{K}$ denotes the sparse weight vector and s is the sparsity level.

Other assumptions for BPFA include the following. (i) Every dictionary atom $d_{k}$, for k∈{1,…,K}, is drawn from a zero‐mean multivariate Gaussian distribution. (ii) Both the components of the noise vectors $n_{p}$ and the non‐zero components of the sparse weight vectors $\alpha_{p}$ are drawn *i.i.d*. from zero‐mean Gaussian distributions. (iii) The sparsity prior on the weight vectors is promoted by the Beta‐Bernoulli process.^11^ Those assumptions can be mathematically modelled in a hierarchical format as

(7a) $$\begin{array}{cccc} z_{p} & =P_{\Omega_{p}}\left(D\alpha_{p}+n_{p}\right),\; & \alpha_{p} & =u_{p}⊙w_{p}\in R^{K}, \end{array}$$

(7b) $$\begin{array}{cccc} D & ={\left[d_{1}^{⊤},\text{…},d_{K}^{⊤}\right]}^{⊤},\; & d_{k} & ∼N\left(0,\gamma_{d}^{-1}I_{N_{\mathrm{op}}}\right), \end{array}$$

(7c) $$\begin{array}{cccc} w_{p} & ∼N\left(0,\gamma_{w}^{-1}I_{K}\right),\; & n_{p} & ∼N\left(0,\gamma_{n}^{-1}I_{N_{\mathrm{op}}}\right), \end{array}$$

(7d) $$\begin{array}{cccc} u_{p} & ∼\;\prod_{k=1}^{K}\mathrm{Bernoulli}\left(\pi_{k}\right),\; & \pi_{k} & ∼\;\mathrm{Beta}\left(\frac{a}{K},\frac{b(K-1)}{K}\right), \end{array}$$

for every patch indexed by $p\in \{1,\text{…},N_{\mathrm{patch}}\}$ and every dictionary atom indexed k∈{1,…,K}. In the equations above, the identity matrix of size K×K is denoted by $I_{K}$, ⊙ denotes the Hadamard product, and a and b are the parameters of the Beta process, $u_{p}$ in is a binary vector controlling which dictionary atoms are used to represent $x_{p}$. The probability of using a dictionary atom $d_{k}$ for representing $x_{p}$ is $\pi_{k}$. Moreover, $\gamma_{d}$, $\gamma_{w}$, and $\gamma_{n}$ are the precision parameters.

BPFA infers all the unknown parameters in the hierarchical model above. However, different methods can also be used for inference, such as, Gibbs sampling^46^ and variational inference.^11^ In this paper, we use a BPFA with Expectation Maximisation (EM)^48^ inference. See Ref. [10] for more details on the BPFA‐EM.

## 4 NUMERICAL RESULTS

**Fully sampled experimental data**. A fully sampled data set – that is the collection of $y_{l}$ for $l\in \{1,\text{…},N_{p}\}$ in Equation 1 ‐ ‐ used for these simulations is a sample of a Ni‐superalloy. Data were acquired on a Zeiss EVO15 SEM equipped with an Oxford Instruments Symmetry S3 detector at an accelerating voltage of 20 KeV, magnification of 1045×, exposure time of 0.9 ms, detector speed of 1057 Hz, and a total acquisition time of 3 min 21 s, with $\left(H_{p},W_{p}\right)=\left(512,416\right)$ probe positions. The EBSPs were binned during acquisition resulting $\left(H_{d},W_{d}\right)=\left(156,128\right)$ detector pixels.

An aperture size of 30 µm and working distance of 22.5 mm, corresponds to a beam convergence angle of 1.33 mrad^8^ which gives a Nyquist sampling rate of 14.2 nm, which is finer than the step size of 481 nm used for this sample. This suggests that this sample is not inherently oversampled. However, it is clear that this sample contains mostly larger grains with few small grains and it is clear that these large grains are oversampled. It is agreed that approximately 10 data points are needed per grain for assessment of grain size, which is surpassed by many of the grains in this sample.^49^

Like all conventional scanning methods, sub‐sampling requires the sampling rate to be tailored to the feature size being observed – this is why microscopes have a range of magnification settings. Small grains will be detected and inpainted if they have been sampled (i.e. if the magnification of the microscope is set to a level to allow them to be sampled). However, it is completely obvious that if the grain is not sampled it will not be inpainted.

**Reference EBSD maps**. The reference band contrast and IPF Z maps, denoted by x in Equation 4 and shown in Figure 2, were generated using Hough‐based indexing in AZtec from the fully sampled data, since this is a fast and readily available method. The indexing parameters used were optimised band detection with 11 bands and a Hough resolution of 40.

No additional postprocessing, for example, cleaning, was performed.

The above EBSD maps were selected due to their differing properties: the band contrast map is a greyscale image and the IPF Z map is a 3D RGB image. There are also other EBSD maps which rely on a full EBSD data set, for example, kernel average misorientation maps where pixels are grouped together to assess misorientation^1^

**Quality criteria**. Given a pair of noisy signal v and noiseless signal u, the Signal‐to‐Noise‐Ratio (SNR) in dB is defined as

(8) $$\mathrm{SNR}=20\log_{10}\frac{∥u∥}{∥u-v∥},$$

where $∥u∥≔(\sum_{i=1}^{N}|u_{i}{{|}^{2})}^{1/2}$ represents the $\ell_{2}$‐norm of u. The robustness of Hough‐based indexing is measured using a Hit Rate (HR) and normalised $\ell_{2}$‐norm error. The hit rate is a measure of the percentage of EBSPs that have been successfully indexed.

(9) $$\mathrm{HR}=1-\frac{|\Omega_{\mathrm{zsp}}|}{N_{p}}.$$

The quality of the reconstructed maps are measured with respect to the reference maps using the Structural Similarity Index Measure (SSIM).^50^

**Noise models**. Two noise models were used in order to test the robustness of the EBSD indexing process. Gaussian noise and Poisson noise both affect microscopy data,^51^, ^52^ with Gaussian noise most commonly encountered as detector readout noise,^51^ while Poisson noise is most encountered as counting noise, from the counting of electrons.^53^

An independent Gaussian noise vector $\eta \in R^{N}$ of size N was generated following an independent and identically distributed (i.i.d.) Gaussian distribution, that is, $\eta_{i}∼N\left(0,\sigma_{\mathrm{gsn}}\right)$ for i∈{1,…,N}, with zero mean and standard deviation $\sigma_{\mathrm{gsn}}$. Therefore, given a noiseless signal y – and considering one index of EBSPs in Equation 1 ‐ ‐ its Gaussian noisy version is generated as

(10) $$y^{\mathrm{gsn}}=y+\eta .$$

Given a desired SNR value in dB, we set $\sigma_{\mathrm{gsn}}$ as

(11) $$\sigma_{\mathrm{gsn}}=\frac{\left\|y\right\|}{\sqrt{N_{p}}}\cdot 10^{-\frac{SNR}{20}}.$$

Poisson noise is important in electron microscopy for counting direct electron detectors. The number of counts at a given detector pixel location is proportional to the scattering cross‐section associated with that scattering angle,^54^, ^55^ which can be modelled by a Poisson distribution.

For this case let $y^{\mathrm{psn}}\in R^{N}$ be a noisy version of the noiseless signal y corrupted by Poisson noise. Therefore, $y^{\mathrm{psn}}$ is a random vector of N random variables with Poisson distribution, that is, $y_{i}^{\mathrm{psn}}=P\left(y_{i}\right)$ for i∈{1,…,N}. However, given a desired SNR value in dB, we can generate a noisy signal following

(12) $$y_{i}^{\mathrm{psn}}=P\left(\sigma_{\mathrm{psn}}\frac{y_{i}}{{∥y∥}_{1}}\right),$$

where ${∥u∥}_{1}≔\sum_{i=1}^{N}\left|u_{i}\right|$ is the $\ell_{1}$‐norm of vector $u\in R^{N}$ and $\sigma_{\mathrm{psn}}$ controls the total absolute intensity of the noiseless signal. Assuming the vector y as one instance of EBSPs in Equation 1, $\sigma_{\mathrm{psn}}$ models the total number of scattered electrons. A higher $\sigma_{\mathrm{psn}}$ corresponds to more electrons striking the detector. In our simulations, given a desired SNR we set $\sigma_{\mathrm{psn}}$ such that the resultant SNR is close to the desired SNR, following

(13) $$\sigma_{\mathrm{psn}}=\frac{{\left\|y\right\|}_{1}^{2}}{{∥y∥}^{2}}\cdot 10^{\frac{SNR}{10}}.$$

### 4.1 Robustness of indexing to noisy data

**FIGURE 4:**
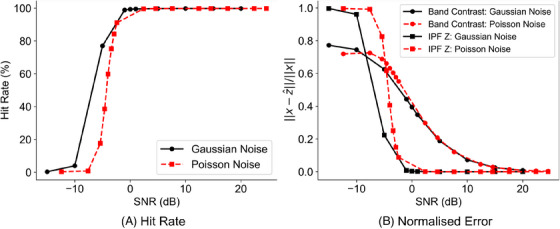
Effect of Gaussian and Poisson noise models on Hough‐based indexing. (A) Hit rate; (B) normalised error between reference and indexed EBSD maps. Due to the high amount of redundancy in EBSD data, the indexing process is robust to moderate noise levels. Data points from 5 and –5 dB are shown in Figure 5.

In this section, Monte Carlo simulations are described with Gaussian and Poisson noise models for multiple independent realisations of noise with input SNR values in the range of [−5,5] dB. Noisy data sets were reprocessed using Hough‐based indexing in the AZtec software. From this, the measured hit rate was recorded and plotted, as shown in Figure 4A. This curve shows that for both noise models the hit rate remains above 99.5% for input SNR>0 dB. Note that for input SNR=0 dB, signal and noise powers are equal.

The normalised error between the reference and indexed EBSD maps is shown in Figure 4B.

For the band contrast map, the normalised error slowly decreases for both noise models. In comparison, for the IPF Z map, the normalised error has a sharp drop when the SNR is between –8 dB and 0 dB. Those observations indicate the indexing processes involved for generating band contrast and IPF Z maps are robust to both Gaussian and Poisson noise models and yield high‐quality EBSD maps for moderate noise levels, that is, SNR values larger than 10 dB.

**FIGURE 5:**
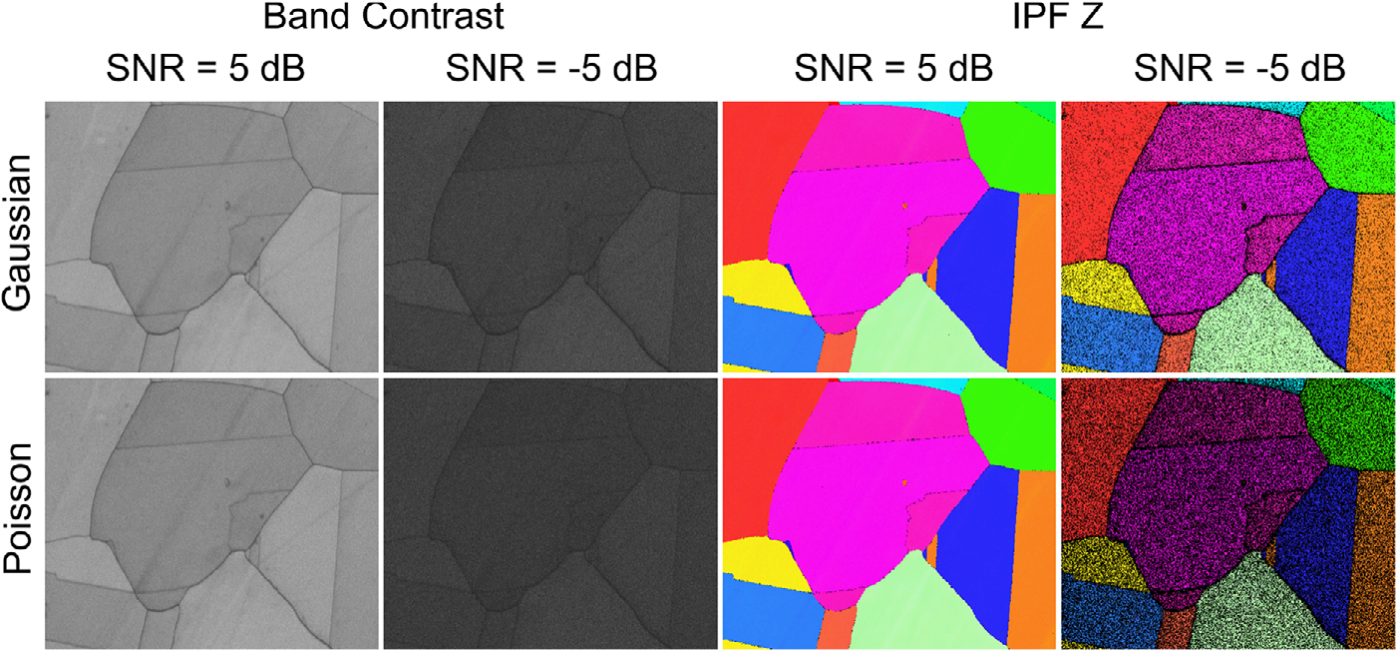
Band contrast and IPF Z maps produced through Hough‐based indexing of noisy EBSD data corrupted by Gaussian and Poisson noise. The indexing quality of these maps are shown in Figure 4.

Examples of the EBSD maps associated with the SNR values of 5 dB and –5 dB are shown in Figure 5, where a lower hit rate is evident in the IPF Z map associated with the SNR = –5 dB, with more ZSPs being present across the map. For the same noise level, the band contrast map suffers from low contrast.

### 4.2 Improving the robustness of IPF maps to strong noise

From the definition of ZSPs, and the observation in Figure 4, our goal in this section is to correct the ZSPs in the IPF maps. We consider ZSPs as unsampled pixels; hence, the associated subsampled IPF map can be inpainted. That approach is referred to as ZSP correction. We emphasise that the subsampling of the IPF map here is not due to the probe subsampling. We will investigate that scenario momentarily.

**FIGURE 6:**
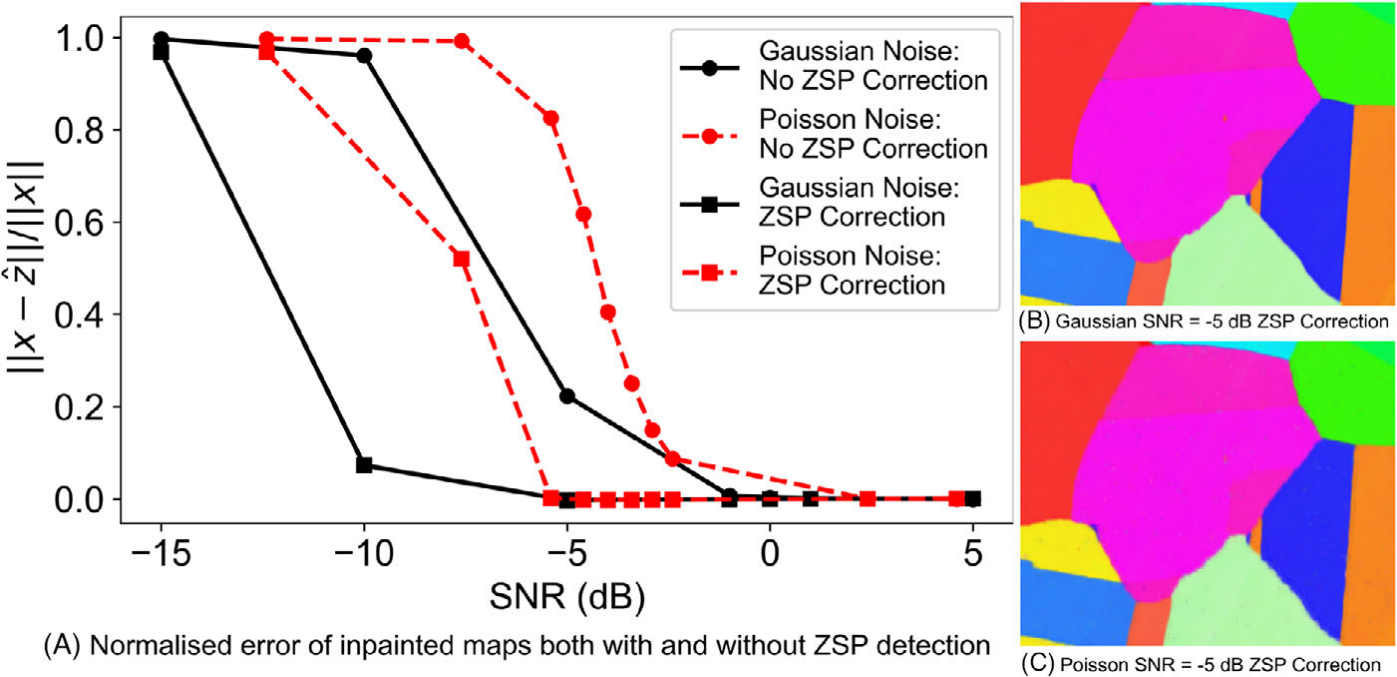
(A) Normalised error of inpainted maps both with and without ZSP detection. (B, C) IPF Z maps with ZSP correction. Examples of these maps without ZSP correction can be found in Figure 5.

Starting from Figure 4, we limit the range of investigated SNR values to only a range where the hit rate is lower than 99%. A comparison of normalised error of inpainted data sets with SNR∈[−15,5] dB for both noise models, with and without ZSP correction is shown in Figure 6a. We observe that ZSP correction significantly improves the indexing error, for example, a 0.97 relative error improvement for Gaussian noise with SNR=−10 dB and a 0.98 relative error improvement for Poisson noise with SNR=−7.6. Examples of the inpainted maps corresponding to SNR = –5 dB are shown in Figure 6b and c. Those two maps should be compared with their counterparts in Figure 5 with the same SNR values. It is evident that the maps from ZSP correction are fully inpainted; the hit rate is now 100% and shows higher quality when compared to the reference map.

### 4.3 Robustness of proposed method to subsampled noisy data

In this part, we simulate a postindexing reconstruction framework as shown in Figure 3 with both noisy and subsampled EBSD data sets generated, then indexed, and finally inpainted. Following a UDS strategy of probe positions, subsampling rates $M_{p}/N_{p}\in \left\{1,5,10,15,20,25\right\}\%$ were investigated in conjunction with two input SNR values, that is, SNR∈{−5,5} dB for both Gaussian and Poisson noise models. For comparison, we have also included a noiseless case.

For computational reasons, band contrast maps were normalised to the range of [0, 255], a typical pixel range for an 8‐bit image, prior to inpainting and subsequently renormalised to their original range afterward.

Following the success of inpainting for correcting the ZSPs in the IPF map in Section 4.2, those maps had an additional step performed before inpainting to identify the ZSPs, as explained previously. Here we do not include the zero solution pixels in the calculation of subsampling rate, therefore, the effective subsampling rate – that is, that used by the inpainting algorithm – corresponding to IPF map simulations is lower than the desired subsampling rate set according to $M_{p}/N_{p}$ ratios mentioned above. More precisely, the effective subsampling rate will be in the range of $\left[\frac{|\Omega_{\mathrm{zsp}}|+M_{p}}{N_{p}},\frac{M_{p}}{N_{p}}\right]$. A previous work^56^ did not include this step, resulting in untreated ZSPs, which in turn limited the reconstruction quality.

Although it is not necessary for the sample to be fully indexed to be measured, the method of detecting zero solution pixels aids in the accuracy of the inpainting. In this way, this method is similar to a cleaning method, with the missing information being learned from the information available from the sampled points.

Effective inpainting parameters for the BPFA algorithm were selected based on a parameter optimisation routine consisting of a series of full factorial design of experiment trials. The common parameters for all simulations were as follows: dictionary atoms K=25, sparsity limit s=4, batch size of 1024, and only one epoch, that is, one pass over the full data set.

It has been previously demonstrated that patch shape has the greatest effect on the outcome of a BPFA reconstruction.^12^ Therefore, the patch shapes were selected individually for each subsampling rate and each EBSD map type. These are shown in Table 1.

**TABLE 1:** Patch shape selected at each sampling rate for inpainting.

	**Patch shape** $\left[H_{\mathrm{op}},W_{\mathrm{op}}\right]$	
**Sampling rate** (%)	**band contrast**	**IPF Z**
25	[6, 6]	[9, 9]
20	[8, 8]	[11, 11]
15	[8, 8]	[11, 11]
10	[10, 10]	[13, 13]
5	[16, 16]	[14, 14]
1	[27, 27]	[23, 23]

**FIGURE 7:**
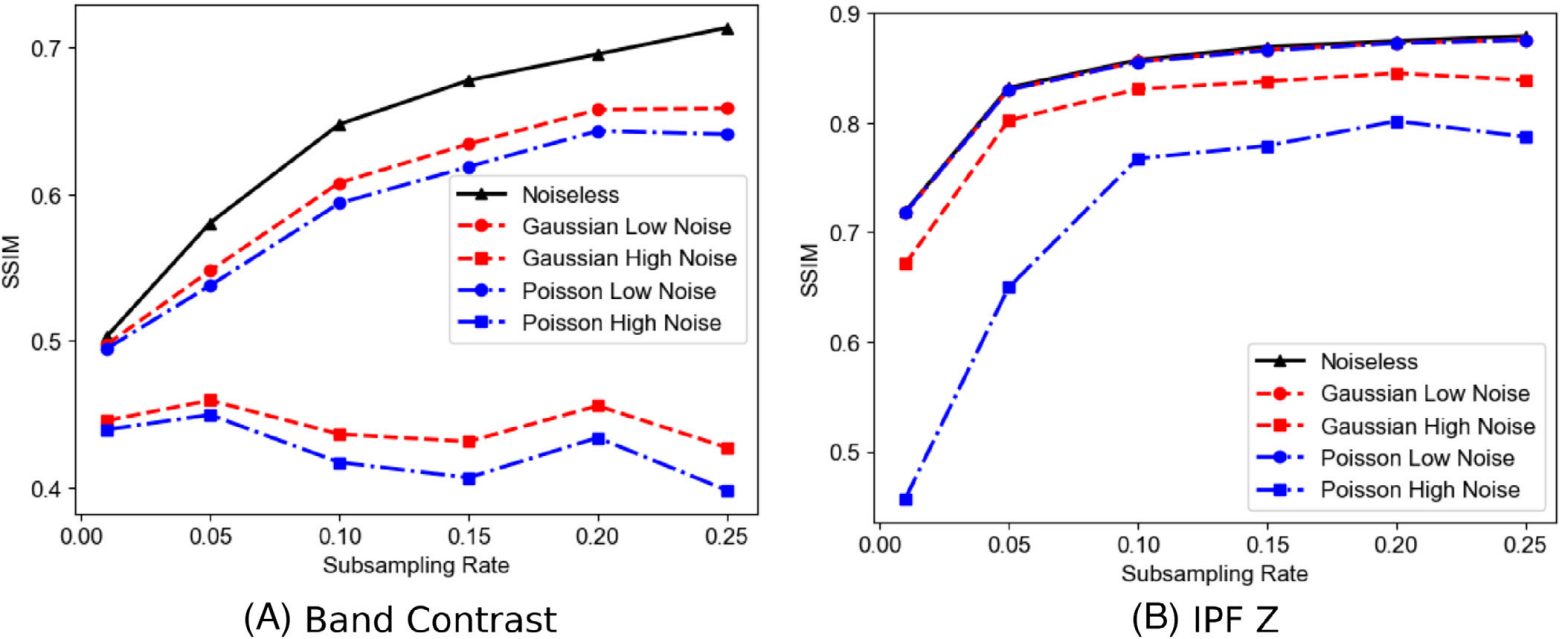
Image quality of reconstructed subsampled EBSD maps showing the effects of noise on the quality of the images reconstructed. (A) Band contrast; (B) IPF Z maps.

The inpainting results are shown in Figure 7 for the band contrast maps. the inpainting results show a steady increase from 1% to 10%, which then plateaus as the subsampling rate increases further. The same trend is found for the noisy data sets when SNR=5 dB, for both noise models. The plateau suggests that it should be possible to subsample EBSD data sets down to 10%, with band contrast maps reconstructed with a quality comparable to that achieved from a full EBSD data set. It is noted that the band contrast SSIM results are lower than the IPF Z results since the band contrast maps have ‘noise’ between pixels. A comparison between subsampled maps and maps inpainted with 100% sampling in order to reduce this noise are shown in Figure S1. However, for input SNR=−5 dB, there is a significant detrimental impact on the inpainting results, which was expected from the data in Figure 4: indexing an EBSD data set with such strong noise gives a normalised error of approximately 0.7.

The IPF Z results are shown in Figure 7. An immediate observation is that inpainting IPF maps is more robust to noisy and subsampled data compared to that of band contrast maps for which the plateau starts at 5% subsampling rate. The reason for this is twofold. First, an IPF map is a three‐colour map with more diversity; hence, helping the inpainting algorithm improve the reconstruction quality. Secondly, ZSPs are considered as unsampled probe positions. Therefore, those pixels are corrected during inpainting and the SSIM values for the clean data set and the data set with SNR=5 dB are almost identical. For these maps a lower SSIM than the band contrast maps is obtained at a very low subsampling ratio of 1%. The proposed method shows reasonable robustness to a very strong Gaussian noise (SNR=−5 dB), with its SSIM curve slightly shifted down. However, a data set corrupted by a very strong Poisson noise (SNR=−5 dB) gives lower SSIM values, which is likely due to its lower hit rate (39% compared to 77% for the Gaussian noise) as shown in Figure 4. Overall, those results suggest that subsampling only 10% of probe positions and inpainting the missing data in the band contrast and IPF maps yields high‐quality maps, even if the data is corrupted by a moderate Gaussian or Poisson noise, that is with SNR=5 dB.

**FIGURE 8:**
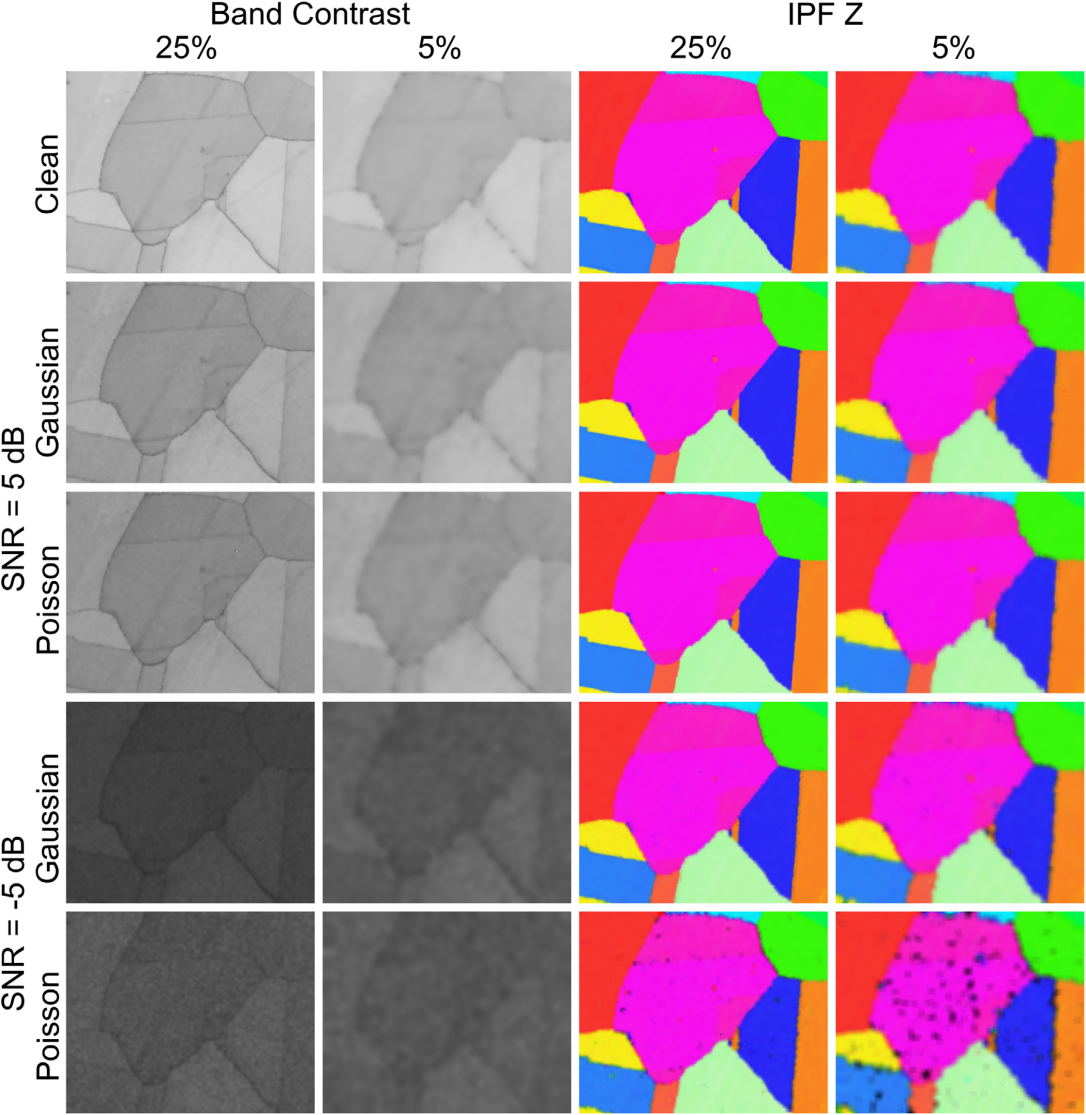
Examples of inpainted band contrast and IPF Z maps. These datapoints are taken from Figure 7.

Examples of the inpainted clean and noisy band contrast and IPF Z maps are shown in Figure 8. The effect of the lower subsampling rate is evident with more blurring being present at 5% than at 25%. The effect of noise is also seen in the band contrast maps, with a darker map being produced by noisier data sets.

### 4.4 Subsampling versus coarser sampling

**FIGURE 9:**
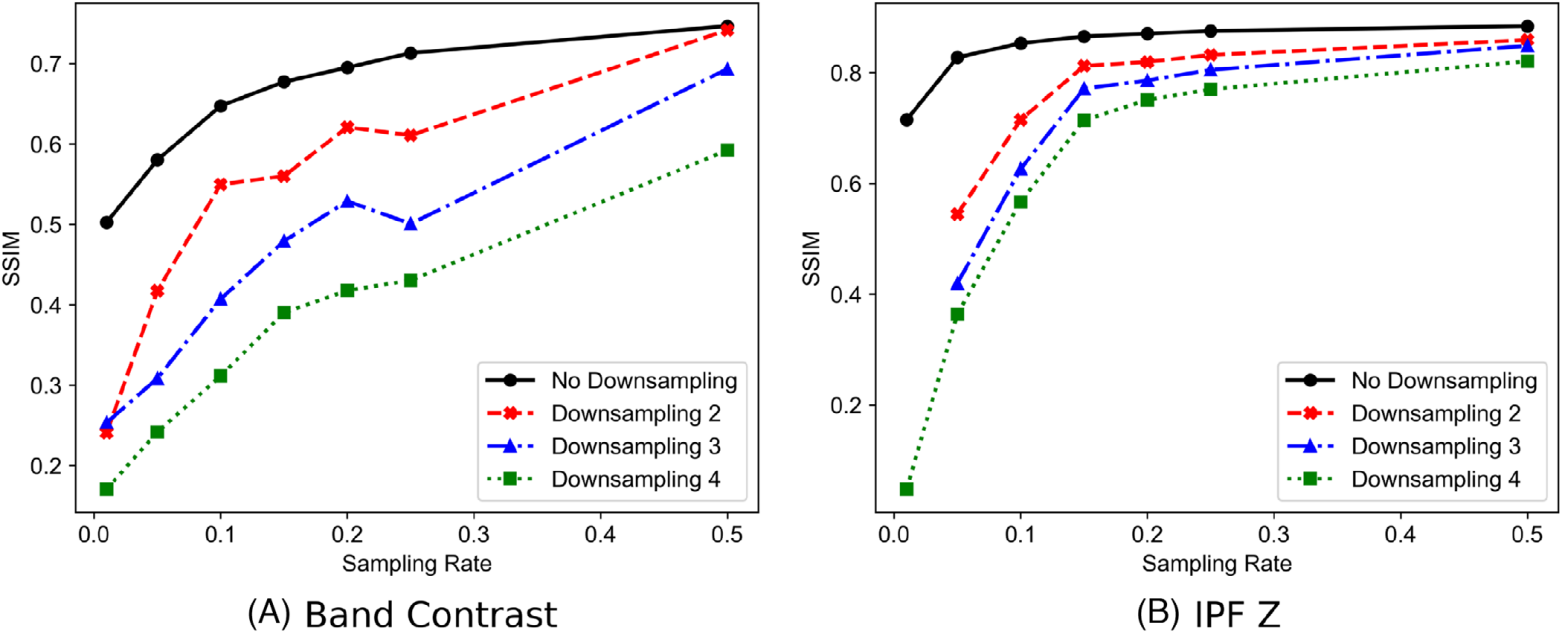
SSIM results for band contrast and IPF Z maps with subsampling in comparison to those which have been subsampled and downsampled.

The use of a coarser grid is also a valid method for improving the speed of acquisition and has also been compared to subsampling. The application of coarser grids was simulated by downsampling the images by factors of 2, 3 and 4, giving effective sampling rates of 25%, 11.1% and 6.25%. The reconstructed image quality is shown in Figure 9. These results show that subsampling is also effective when a coarser sampling grid is applied through the plateau of the curves. By subsampling images on a coarser grid significant speed improvements can be realised this could be useful, for example, in identifying areas of interest or for analysing large areas of the sample for fast measurements.

## 5 CONCLUSION AND DISCUSSION

We have presented a proof of concept for compressive EBSD imaging. We show the efficacy of subsampling and inpainting applied to both clean and noisy EBSD data sets.

Using BPFA as an inpainting method, clean data sets can be subsampled to 10%, with the potential to reduce this to 5% if only IPF Z maps will be are required. Data sets with moderate noise levels have a similar robustness to subsampling, with the band contrast showing robustness to 10% subsampling and IPF Z to 5% subsampling. Overall, we have shown that ZSP correction via inpainting enables higher quality reconstructions of IPF maps when the EBSD data set is corrupted by strong noise.

Compared to the proposed method based on postindexing reconstruction, an alternative mechanism would be based on preindexing reconstruction involving inpainting the EBSPs, forming a complete 4D EBSD data set for indexing. This alternative mechanism of reconstruction is expected to be a more robust method of inpainting EBSD data sets, allowing fast and low dose acquisition suitable for more beam sensitive samples. Furthermore, leveraging the high amount of diversity in the 4D EBSD data set during the inpainting process would enable noisier data sets, equivalent to lower electron dose, to be collected.

These results show how subsampling can be applied to EBSD data sets. Whilst these results are positive, further work to ensure robustness is necessary. These include but are not limited to experimentally noisy data sets; small grains and defects/deformations.

Although this method is robust to the noise models shown here, its equivalency to experimental noise will be investigated. Experimental noise tends to result in greater noise levels around the grain boundaries reflected by more ZSPs. This should not prevent the inpainting algorithm from being effective, although it could reduce the resolution at grain boundaries. As shown earlier, there is blur in the reconstructions which may increase where less information is available. Alternatively, experimentally noisy data sets can result in mis‐indexed pixels. Although it's possible that the inpainting algorithm could remove low levels of mis‐indexing, where many mis‐indexed pixels are present the method will be less robust, since the learned information will be inaccurate. It is anticipated that for these types of data sets, reconstructing the full data set through pre‐indexing reconstruction will be more robust, since the EBSPs will be subject to denoising.

Although the data set shown here contains mostly larger grains, the small grains reconstructed in the EBSD map show that the algorithm has the sensitivty to small grains. Where small grains are present the most important aspect of this method is the selection of sampling rate, since any small grains that are not sampled will not be reconstructed. This method will be investigated on further samples, notably geological samples containing multiple phases, as well as an alternative inpainting method such as preindexing reconstruction to determine the best methods for sensitivity and speed.

Samples containing deformations or defects will also be investigated.

High angular resolution EBSD is one specific technique that could benefit from proposed framework. High angular resolution EBSD requires high resolution EBSPs in order to measure acute angles between expected and experimental bands in EBSPs.^57^ These high resolution EBSPs therefore have long acquisition times. By subsampling probe positions significant time could be saved during the acquisition.

Similarly, 3D EBSD is another technique with lengthy acquisition times. In 3D EBSD layers of a thick sample are cut, with a full EBSD map being acquired at each layer.^57^ That technique is limited by both cutting time and acquisition time, which could be significantly decreased through an application of probe position or layer subsampling. In that context, targeted sampling, which has been previously demonstrated in Ref. [27], would be a potential method for locating regions of interest such as grain boundaries.

## Supporting information

Supporting Information
