# Supplementary material for: Compressive electron backscatter diffraction imaging
Source: J Microsc. 2025 Jan 11;298(1):44–57. doi: 10.1111/jmi.13379 (PMC11891967; doi:10.1111/jmi.13379)
Supplement: Supplementary file 1 — Supporting Information [file JMI-298-44-s001.pdf]

Compressive Electron Backscatter Diffraction Imaging - Supplementary Information

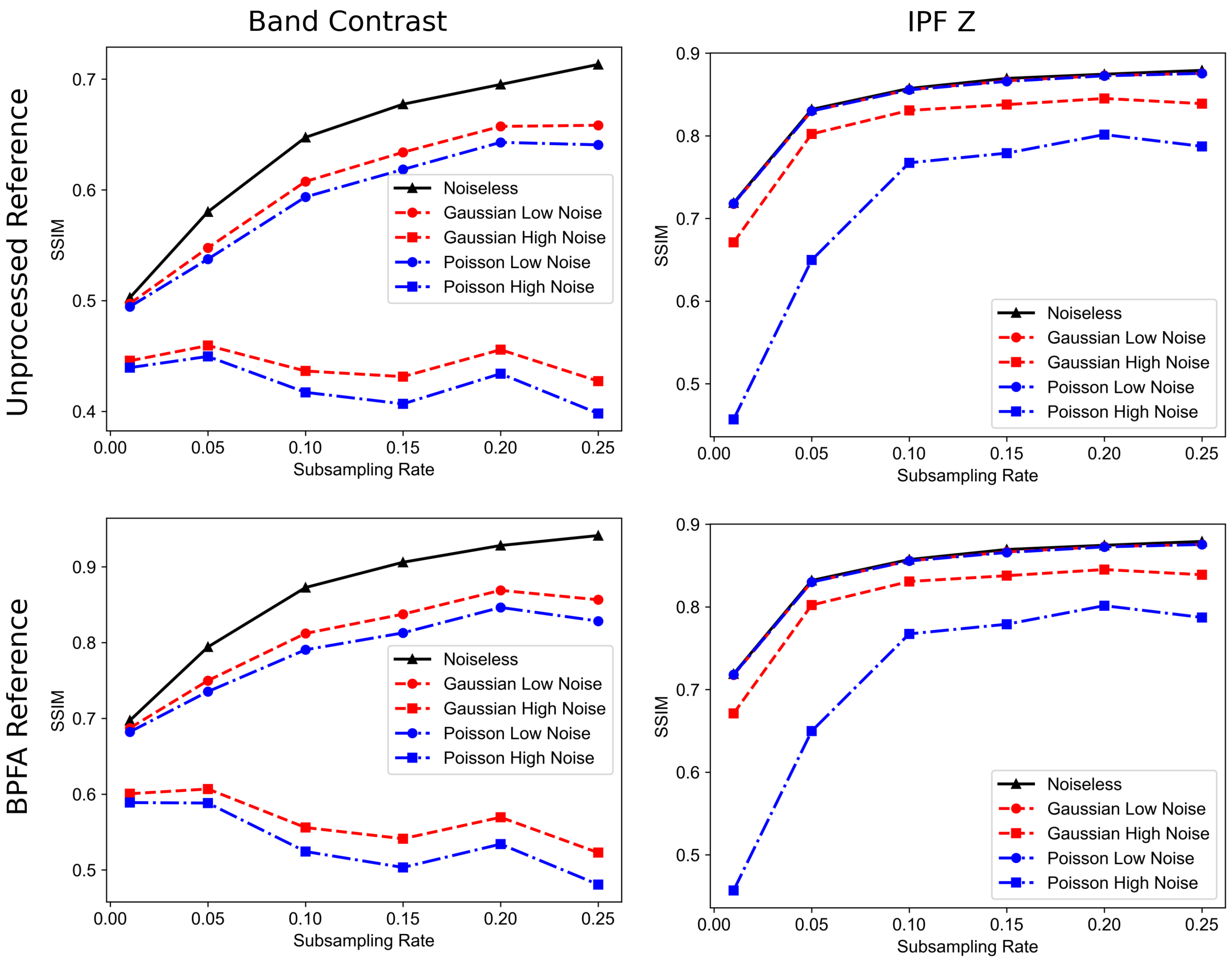

Fig. 1: Comparison of SSIM results for both band contrast and IPF Z maps using both an unprocessed reference and a 100% inpainted reference.
In band contrast maps, the pixel value is based off a single EBSP. Since the band contrast varies between each EBSP, this results in variations between the pixel values, similar to a noisy image. The inpainted reference shows an improvement in the reconstruction quality since it removes the noise present in the band contrast maps. This noise is not present in the IPF Z maps since the pixels values are based on crystallographic orientation rather than the contrast in the EBSP.
